# P02.103. Banha-sasim-tang, a herbal formula, for the treatment of functional dyspepsia: a randomized, double-blind, placebo-controlled, two-center trial

**DOI:** 10.1186/1472-6882-12-S1-P159

**Published:** 2012-06-12

**Authors:** J Park, S Ko, G Han, B Ryu, I Yeo, J Kim

**Affiliations:** 1 College of Oriental Medicine, Kyung Hee University, Seoul, Republic of Korea; 2 Department of Statistics, College of Sookmyung Women's University, Seoul, Republic of Korea

## Purpose

Functional dyspepsia (FD) is a chronic gastrointestinal disorder characterized by dyspeptic symptoms without structural lesions. There are no standard treatments for FD until now. Thus, many researchers had been interested in alternative therapies, including herbal formulas, for FD treatment. However, alternative therapies need more evidence. In traditional Korean medicine, Banha-sasim-tang (BST) is a famous herbal formula for treating dyspepsia and there is no relevant study of FD using BST. The aims of the trial are to investigate the efficacy and safety of BST for FD and to evaluate the relationship between dyspepsia and cutaneous electrogastrography (EGG).

## Methods

This randomized, double-blind, placebo-controlled trial at two medical centers in Korea consisted of BST and placebo groups (n=50, respectively). BST or placebo was administered for 6 weeks to FD patients and subsequent 2-month follow-up observation was conducted. Primary outcome was the difference as measured on the gastrointestinal symptom (GIS) scale, and secondary outcomes were differences as measured on the visual analogue scale (VAS) for dyspepsia and on the questionnaire for FD-related quality of life (FDQoL). All outcomes were assessed at baseline, 2, 4, and 6 weeks of treatment, and at 1 and 2 month follow-up. EGG was performed at baseline and 6 weeks.

## Results

GIS, VAS and FDQoL insignificantly decreased in both BST and placebo groups. However, early satiety significantly improved in BST groups (p<0.05). Percentage distribution of tachygastria among post-prandial dominant power values in channel 3, which might be related to pyloric dysmotility, significantly decreased after BST administration (p<0.05).

## Conclusion

BST did not show a significant effect on FD. However, early satiety among various dyspeptic symptoms and tachygastria around pylorus from cutaneous EGG measurement might be improved throughout BST administration. Further studies focused on mechanisms related to gastric motility modulation will be needed for more relevant clinical applications to FD.

